# Manipulating carer number versus brood size: complementary but not equivalent ways of quantifying carer effects on offspring

**DOI:** 10.1093/beheco/arw038

**Published:** 2016-03-28

**Authors:** A.L. Liebl, L.E. Browning, A.F. Russell

**Affiliations:** 1 ^a^ Centre for Ecology & Conservation, College of Life & Environmental Sciences, University of Exeter, Treliever Road, Penryn, Cornwall TR10 9FE, UK and; 2 ^b^ UNSW Arid Zone Research Station, School of Biological, Earth & Environmental Sciences, University of New South Wales, Sydney, New South Wales 2052, Australia

**Keywords:** cooperative breeding, disruption hypothesis, helper effect, scramble competition, territory quality.

## Abstract

Experiments designed to quantify the effects of increasing numbers of carers on levels of offspring care are rare in cooperative breeding systems, where offspring are reared by individuals additional to the breeding pair. This paucity might stem from disagreement over the most appropriate manipulations necessary to elucidate these effects. Here, we perform both carer removal and brood enhancement experiments to test the effects of numbers of carers and carer:offspring ratios on provisioning rates in the cooperatively breeding chestnut-crowned babbler (*Pomatostomus ruficeps*). Removing carers caused linear reductions in overall brood provisioning rates. Further analyses failed to provide evidence that this effect was influenced by territory quality or disruption of group dynamics stemming from the removals. Likewise, adding nestlings to broods caused linear increases in brood provisioning rates, suggesting carers are responsive to increasing offspring demand. However, the 2 experiments did not generate quantitatively equivalent results: Each nestling received more food following brood size manipulation than carer removal, despite comparable carer:offspring ratios in each. Following an at-hatching split-design cross-fostering manipulation to break any links between prehatching maternal effects and posthatching begging patterns, we found that begging intensity increased in larger broods after controlling for metrics of hunger. These findings suggest that manipulation of brood size can, in itself, influence nestling provisioning rates when begging intensity is affected by scramble competition. We highlight that carer number and brood size manipulations are complimentary but not equivalent; adopting both can yield greater overall insight into carer effects in cooperative breeding systems.

## Lay Summary

Measuring the causal effects of increasing carer number on offspring success is required to understand the evolution of cooperative care systems. Here, we did so using 2 experimental techniques in the chestnut-crowned babbler from outback Australia. Both carer removal and brood size manipulations indicate causal effects of helpers on offspring food acquisition. However, the results were not equivalent, with nestlings receiving more food following brood size manipulations, even after controlling for similar carer to offspring ratios.

## INTRODUCTION

The level of sustenance received by offspring during development has significant impacts on their survival and subsequent reproductive potential (Lindström 1999). Although mothers are responsible for providing most resources prebirth, in biparental and cooperative breeding systems, putative fathers and nonbreeding helpers can add to maternal provisioning postbirth (Harrison et al. 2009; Russell and Lummaa 2009). Quantifying the impacts of such additional carers on offspring provisioning rates will yield important insights into the evolution of such cooperative care systems. In biparental care systems, a meta-analysis of experimental studies confirms putative fathers have additive effects on brood provisioning rates because increases in their contributions are generally met with only partial reductions by their partner (Harrison et al. 2009). However, this meta-analysis also revealed that the type of manipulation performed influences rates of food acquisition by the brood. For example, fathers provisioned more when their partner’s rate was reduced through tail-weighting, but not flight feather-clipping, despite an equivalent reduction in maternal care under both manipulations (Harrison et al. 2009). The degree to which offspring benefit from the addition of more carers in cooperative breeding systems has been subject to relatively few experimental tests, and fewer still have clarified whether increasing carer number positively influences the level of sustenance received by offspring. This may be in part due to the fact that there is also disagreement over the type of manipulation that should be performed to quantify the impact of increasing carer number on offspring success in cooperative breeders (Cockburn 1998), and no study has compared candidate methods.

Initially, the impact of increasing carer number on breeding success was assessed experimentally in cooperative breeders by measuring the consequences of removing nonbreeding helpers. In the first such study, Brown et al. (1982) permanently removed all nonbreeding helpers, save one, from groups of gray-crowned babblers (*Pomatostomus temporalis*) and found experimentally created trios subsequently reared similar numbers of fledglings in the season to naturally occurring trios, but 3 times fewer than larger control groups. This effect was driven by the effect of helpers on the number of further breeding attempts in a season, but whether or not carers also improved nesting success through provisioning was not clarified. Similarly, Komdeur (1994) demonstrated 70% reduced fledging success following translocation of helpers from cooperative groups of Seychelles warbler (*Acrocephalus sechellensis*), but again the degree to which this effect was driven by reduced food acquisition was not investigated. Indeed, only 3 previous studies have collected provisioning data during removal experiments. In Florida scrub-jays (*Aphelocoma coerulescens*), Mumme (1992) showed that control groups fledged 33% more nestlings than groups from which helpers had been removed for the season. In this case, the reduced success following helper removal was mediated through a combination of reduced food provisioning and protection from nest predators. Using short-term (3–8h) removals of helpers in cooperative groups of long-tailed tits (*Aegithalos caudatus*), Hatchwell and Russell (1996) showed that helpers primarily allow breeders to reduce their provisioning rate rather than provide additional sustenance to the brood (but see Hatchwell et al. 2004). Finally, in chestnut-crowned babblers (*Pomatostomus ruficeps*), we have shown that the provisioning rates of male carers are insensitive to temporary carer removals, and so the contributions of such carers to broods are fully additive (Liebl et al. 2016). Although these studies suggest that helpers are beneficial, removal experiments have been criticized as a means to quantifying causal impacts of helpers (Cockburn 1998). The primary reason for this is that reductions in success following helper removal are also expected if the removal of carers disrupts group foraging dynamics and/or established dominance hierarchies (Cockburn 1998).

To avoid the potentially confounding impacts of removals, temporary manipulation of offspring numbers has been advocated as a more satisfactory test of the benefits of additional carers in cooperative breeders. By temporarily translocating meerkat (*Suricata suricatta*) pups from groups, Clutton-Brock et al. (2001) showed that reduced carer:pup ratios (generated by adding pups) led to reduced weight gain in both pups and helpers, whereas increasing carer:pup ratios (by removing pups) led to increased weight gain in pups but not helpers. Although not measured in the experiment, this effect presumably arose because each pup receives more food with increasing carer:pups ratios (Clutton-Brock et al. 2001). Reciprocal brood size manipulations in superb fairy-wrens (*Malurus cyaneus*), demonstrated that the rate at which nestlings received food was a function of carer:nestling ratios rather than group size per se (Russell et al. 2008), suggesting that offspring receive more food with the addition of carers. Finally, in acorn woodpeckers (*Melanerpes formicivorus*), experimentally enlarged broods were fed significantly more frequently than reduced broods, although feeding rates per nestling were greater in the latter (Koenig and Walters 2012). These studies suggest that helpers are responsive to offspring demand and by extension presumably have a causal impact on offspring success.

Although the 2 experimental techniques outlined above are both used to test the causal impacts of helpers on brood and group success, whether or not the results derived from each are equivalent has not been tested. Removal experiments obviously offer a more direct means of quantifying the effects of carer numbers on offspring provisioning, but only if removing carers does not introduce confounding influences on provisioning (Cockburn 1998). By contrast, although manipulations of brood size are suggested to circumvent possible issues arising from the removal of carers, they cannot clarify any potential confounding influences of territory quality and might introduce their own confounding influences if the begging signals to which carers respond to are determined by scramble competition in addition to immediate offspring nutritional requirements (Royle et al. 2002). In short, determining the influence of additional carers in cooperative breeding systems might require a multipronged experimental approach in which the potential confounding consequences of the multiple methods used are also tested.

Here, we use both temporary carer removal and brood size enhancement experiments in the cooperatively breeding chestnut-crowned babbler to quantify the causal impact of carer number on offspring provisioning rates. First, we use carer removal experiments to test for causal effects of carer numbers on nestling provisioning rates and brood size enhancement experiments to test whether carers are responsive to changes in brood demand. Second, we compare whether the 2 methods generate equivalent relationships between carer:offspring ratios and the average provisioning rate of each nestling. This comparison is facilitated by our equalizing of carer:nestling ratios in the 2 experiments. Third, we elucidate the mechanism underlying the differences found between the 2 experimental techniques; in particular, whether or not disruptive effects of carer removals or increased scramble competition induced by brood size enhancements are most likely to drive the differences.

## METHODS

The study was conducted during the breeding seasons of 2007, 2008, 2013, and 2014 at Fowlers Gap Arid Zone Research Station in far western New South Wales, Australia (141°43′E, 31°05′S). Fieldwork was carried out under the approval of Macquarie University Animal Care and Ethics Committee (license no. 06/40A), the NSW National Parks and Wildlife Service, and the Australian Bat and Bird Banding Scheme. Details of the arid zone climate and babbler socioecology are provided elsewhere (Portelli et al. 2009; Sorato et al. 2012; Russell 2016). Briefly, chestnut-crowned babblers are a 50-g endemic bird of inland regions of south-eastern Australia. Breeding units consist of a single breeding female, 1–4 male breeders (mode = 1), and up to 13 nonbreeding helpers (mean = 4). Increased carer numbers are associated with reduced nestling starvation and increased probability of being multibrooded (Russell et al. 2010; Browning, Patrick, et al. 2012). The principal provisioners are male (fathers and nonbreeders related to the brood by at least second order); breeding females contribute significantly less to provisioning overall and reduce their contributions as the number of carers increase (Browning, Young, et al. 2012). Prey items are always delivered singly, and provisioning rates explain the majority of estimates of biomass delivered to the brood (Browning, Young, et al. 2012). All experiments were conducted within natural levels of variation (Table 1).

**Table 1:** Summary of brood sizes, male carer numbers, carer:nestling ratios, and observation times for control and experimental days during the 2 experiments

Parameter	Control days (carer removals)	Control days (brood size enhancements)	Manipulation days (carer removals)	Manipulation days (brood size enhancements)
Brood sizes	3.5±0.9 (2–5)^a^	3.2±1.0 (2–5)	As on control days^a^	4.9±1.1 (3–6)
Numbers of male carers	4.0±1.5 (2–8)	3.5±1.8 (1–7)^b^	2.4±1.4 (1–6)	As on control days^b^
Carer:offspring ratios	1.54±0.53 (0.8–2.5)	1.48±0.60 (0.4–2.3)	1.04±0.44 (0.4–1.75)	0.94±0.33 (0.3–1.5)
Observation time (h)	16.6±6.2 (8.5–27.9)	14.1±3.7 (5.9–19.8)	5.9±3.7 (1.7–14.2)	10.0±2.4 (4.2–14.2)

Values show means ± 1 SD and ranges in parentheses. Carer:nestling ratios include the breeding female, so do not reflect male carers divided by brood sizes. There was no difference on control days in nests used for carer number versus brood size manipulations in terms of brood sizes, numbers of male carers, or carer:offspring ratios. Additionally, carer:offspring ratios were comparable following carer removal and brood size enhancements. Statistical comparisons are presented in Methods.

^a^Brood sizes remain unchanged on control and experimental days of helper removal.

^b^Numbers of male carers remain unchanged on control and experimental days of brood enlargements.

### Removal of male carers

Fifteen breeding units were captured using mist nets, and 1–3 known male carers (mean = 1.7, standard deviation [SD] = 0.6) were removed at random from each group for up to 36h during the nestling period (brood age = 10–21, mean = 16 days of 23-day nestling period). Groups were captured in the morning and given the rest of the day to acclimate to their new group size; observations of provisioning behavior were conducted on the subsequent day (details below). The breeding status of male carers was not known and polyandry is relatively common (Nomano et al. 2015); so both breeding and nonbreeding males were likely removed. Our inability to distinguish between such males is unlikely to influence our results because all those removed were known provisioners, and breeding and nonbreeding provisioners have equivalent provisioning rates across the range of unit sizes observed (Browning, Young, et al. 2012). Before manipulation, units averaged 4 male carers (range = 2–8); more were removed from larger groups so that none was left with fewer than 2 carers (including breeding female). For full details of numbers of male carers, brood sizes, and carer:offspring ratios before and after manipulation, see Table 1.

Removed carers were transported (<10 km) by vehicle in bird bags and maintained in 2×2.5×2 m (l × b × h) aviary compartments where they had access to natural foraging substrate, perches and a roost nest, as well as ad libitum access to water and meal worms (see Engesser et al. 2015 for further details). All birds were released before sunset on the day following capture. Following release, birds are accepted back into their groups without sign of retribution (Nomano et al. 2015).

### Brood size enlargements

Although we did not use the same breeding units for the 2 experiments, on control days, brood sizes (*T*_31_ = −0.72, *P* = 0.47), numbers of male carers (*T*_31_ = 1.07, *P* = 0.29), and carer:offspring ratios (*T*_31_ = −0.26, *P* = 0.80) were not significantly different between the 2 experimental groups (Table 1). Because carer removals obviously generate reduced carer:offspring ratios, to facilitate comparison between the 2 methods, we used brood enlargement experiments to equalize the manipulated carer:offspring ratios across the 2 treatments (Table 1). To this end, we translocated 1–3 nestlings to broods of comparable age (±2 days) (*N* = 18 broods). Manipulations were conducted when broods were similarly aged as in the carer removal experiments (mean = 13 days, range = 8–16). Where possible, and typically, manipulations were conducted the day before observation so that, as with the helper removals, groups generally had several hours to acclimate to their new brood size before provisioning rates were monitored. The numbers of offspring added depended on initial brood size in both original and foster nests, so that enlarged broods never exceeded the maximum of 6 nestlings naturally found in this species and no nest was left without nestlings (i.e., broods of 2 received 1–3 nestlings [mean = 1.6, *n* = 5 broods]; broods of 3 received 1–3 [mean = 1.8, *n* = 6]; broods of 4 received 1–2 [mean = 1.8, *n* = 5]; and broods of 5 received 1 [*n* = 2]). Nestlings were transported between nests by vehicle in a bird bag (above hot water bottles when necessary); chicks were never out of a nest for more than 45min (usually ca. 20min). All nestlings were returned to their original nests following the experiment, facilitated by banding all chicks prior to translocation. Brood size enhancements created carer:offspring ratios equivalent to those generated during the helper removal experiments (*T*_31_ = −0.74, *P* = 0.46; Table 1).

### Monitoring provisioning

Provisioning rates were determined remotely using a passive integrated transponder (PIT)-tag system (Browning, Young, et al. 2012; Young et al. 2013; Nomano et al. 2014). Briefly, all individuals captured in the population (including nestlings) have a 2×12mm Trovan PIT-tag inserted subcutaneously in their flank. When PIT-tagged individuals pass through a camouflaged copper coil fitted to the entrance of their dome-shaped nests, the identity, along with date and time of all nest visits are recorded to an attached Trovan LID 650 decoder. Integrating this system with nest cameras has revealed that we can identity 99% of all independent nest visits by male carers using decoders alone; given the very low non-feeding and false-feeding rates among such carers in this system (<5% of visits; Young et al. 2013), nest visits closely align with provisioning events (Nomano et al. 2014). For a number of reasons, the provisioning rate of the breeding female cannot be determined using PIT-tags alone (Nomano et al. 2014) and so her rate was excluded from all analyses, although her presence was maintained in the carer:offspring ratios. Excluding the provisioning rate of the breeding female from our analyses will not have a significant bearing on the results because evidence from nest cameras indicates that she contributes little overall to provisioning and carers are unresponsive to her provisioning levels (Browning, Young, et al. 2012). This drawback aside, the key advantage of the PIT-tag system is that large amounts of provisioning data can be collected over long periods of time, which is particularly important in species such as babblers that, overall, have relatively low provisioning rates (ca. 3–4 prey items/h).

Provisioning rates were obtained for both a control and experimental day in each experiment. For groups involved in the removal experiment, control data were collected either the day before removals (i.e., day 1; *N* = 8 groups) or the day following carer re-release (i.e., day 4; *N* = 7 groups), with days 2 and 3 representing capture and monitoring days, respectively. Thus, units were provided a day to recover from capture and acclimate to their new unit size before data collection. For those involved in the brood size manipulations, control-day observations were conducted either the day before the addition of nestlings (i.e., day 1; *N* = 13 groups) or the day following the re-establishment of original brood sizes (i.e., day 4; *N* = 5 groups), with day 2 or 3 representing the day of experimental observation. We restricted our collection of provisioning data to broods at an age (8–21 days) when provisioning rates are stable (general linear model [GLM] of age effects on control days after controlling for carer number and brood size: *F*_1,31_ = 0.18, *P* = 0.67, estimate [± standard error, SE] = −0.090±0.21). Furthermore, as control data were collected either immediately before or after experimental data, overall, broods were similarly aged on control versus experimental days. Observation periods on each nest averaged 15h (±5h SD, range = 6–28h) during control days and 8h (±4h SD, range = 2–14h) during experimental days; periods in excess of 12h spanned periods of daylight on consecutive days. To generate a common currency between the 2 treatments, provisioning data were transformed to per capita provisioning rates (i.e., feeds per hour per chick) by dividing the total number of feeds from male carers by the observation time and then by brood size. Including observation time as a covariate had no impact in any model and so was not included in the analyses.

### Brood begging intensity

We have found no evidence in previous studies to suggest that provisioning contributions by male carers are influenced by mechanisms of pay-to-stay (Nomano et al. 2013) or social prestige (Nomano et al. 2015), but significant evidence for a role of kinship (Browning, Patrick, et al. 2012). As a consequence, we hypothesized brood begging intensity may be a significant mediator of carer provisioning rates. Furthermore, as chestnut-crowned babblers breed in dark, domed-nests and nestlings show no obvious color displays when begging, it is likely that acoustic signals represent the primary means of conveying hunger. As such, we used measures of brood begging intensity to assess 2 key questions. First, we verified that begging intensity is sensitive to short-term variation in levels of hunger, and hence would be affected by the experiments conducted. To do so, we investigated the effects of per capita nestling provisioning rates over the previous hour and time since last feed on begging intensity. Second, we then tested whether brood size or carer number additionally explained variation in begging intensity after controlling for these metrics of hunger.

Begging behavior can be confounded by maternal effects (Groothuis and Schwabl 2008), clouding interpretation of any relationships among hunger, carer number, and brood size on begging intensity. In an attempt to break any links between prehatching maternal effects and posthatching begging patterns, we used split-design cross-fostering at hatching (day 0) to generate broods comprising of 2–6 nestlings genetically deriving from different mothers. Only nests without prior nestling mortality were used to avoid including the begging intensity of starving nestlings. Using internal nest cameras with audio recorders (Browning, Young, et al. 2012; Young et al. 2013), we obtained measures of begging intensity during 920 provisioning visits across 20 days of observation for 12 broods aged 3–18 days. During each visit, begging intensity was categorized as none, minimal, median, or maximal referring to no begging (4% of visits), audible begging without postural change (21%), audible begging with open gape (46%), and audible begging with open gape and clear neck straining (30%), respectively.

### Statistical analyses

Analyses were conducted in Genstat (release 17, VSN Rothamsted Experimental Station, UK). First, we used linear regression analyses to investigate the changes in overall brood provisioning rates between control and experimental days as a function of 1) the number of male carers removed and 2) the number of nestlings added. When used with limited numbers of values in independent terms (i.e., here, 1, 2, or 3 carers removed or nestlings added), regression analyses generate valid slope gradients (Lande and Arnold 1983) but not slope significance. To determine the latter, we subsequently conducted permutation tests in which data were shuffled randomly among the independent values (i.e., 1, 2, or 3) while maintaining the number of data points per value in a given analysis (Manly 1997). Doing so 10000 times allowed us to generate a null distribution of regression slopes against which the probability that our observed slope could arise by chance could be compared. To test for a confounding effect of territory quality on responses following carer removal, we used a multiple regression analysis. In this case, natural logarithmic (ln) transformed per capita nestling provisioning rates on experimental days was fitted as the response term and control versus experimental carer numbers were fitted as explanatory variables. If nestling provisioning rates are determined by an autocorrelation between territory quality and numbers of male carers, we would expect a significant impact of control carer numbers in this analysis.

Second, to investigate whether the 2 experimental methods yielded equivalent results, we fitted (ln-transformed) per capita nestling provisioning rates on experimental days as the response term in a GLM, with experimental method (helper removal vs. brood enhancement) and carer:nestling ratio fitted as interacting explanatory terms. Third, to investigate whether differences between the experiments were driven by the carer removal or the brood enhancements, we compared the slopes of the relationships between carer:nestling ratios and per capita nestling provisioning rates on experimental versus control days in each experiment. In this case, we conducted 2 residual maximum likelihood models (REML) (one for each experiment), in which ln-transformed per capita nestling provisioning rates were fitted as response terms, treatment (control vs. experimental day) and carer:nestling ratio were fitted as interacting explanatory terms, and breeding unit was fitted as a random term to block control and experimental data by breeding unit.

Finally, because we only found evidence to suggest that the brood size manipulation influenced per capita nestling provisioning rates, we investigated the effects of brood size on brood begging intensity. Here, we fitted whether or not carers encountered a maximally begging brood as a binary response term with logit link function in a generalized linear mixed model (GLMM). The rate at which broods received food over the previous hour and the time between the previous and current feed were fitted as covariates to control for brood hunger levels, while carer number and brood size were fitted as fixed effects. Breeding unit identity was fitted as a random term.

## RESULTS

### Causal carer effects

**Figure 1:**
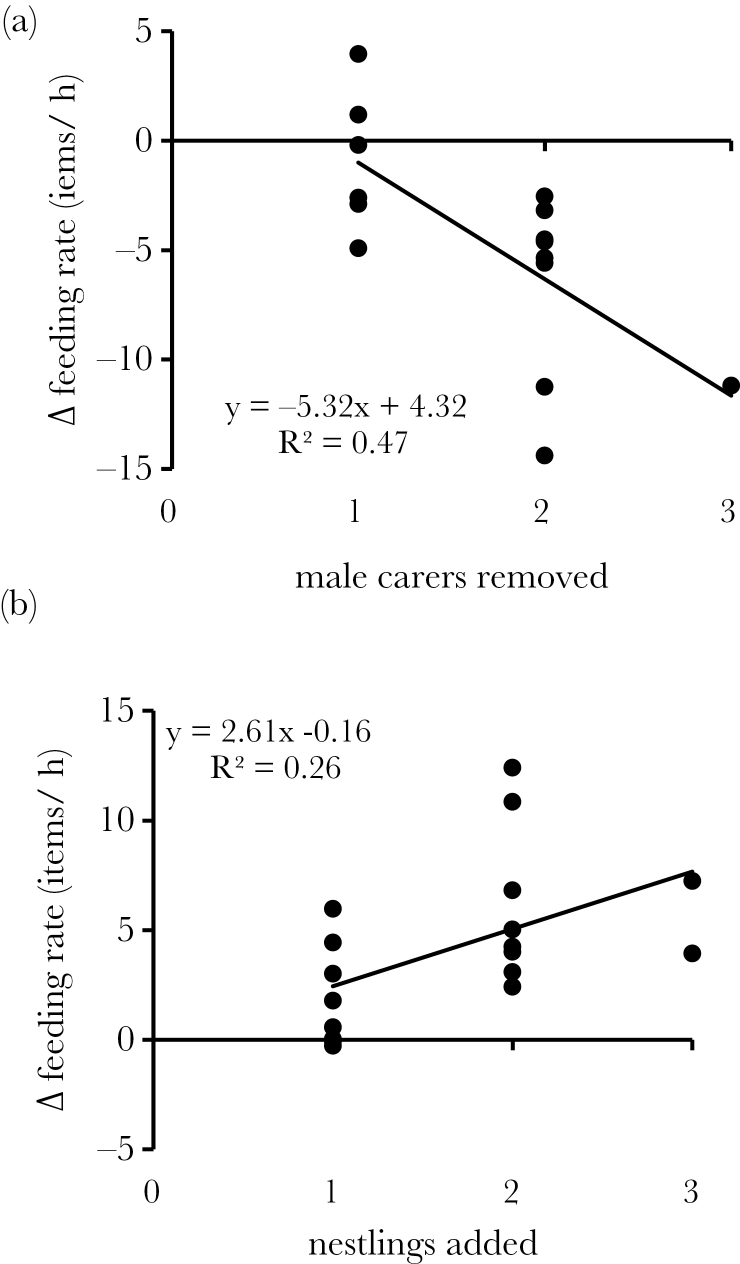
Effects of manipulations on changes in brood provisioning rates. On experimental days, the temporary removal of 1–3 male carers (a) was associated with linear declines in brood provisioning rates, whereas the temporary addition of 1–3 nestlings (b) was associated with linear increases in brood provisioning rates. Figures show raw data and best-fit functions.

On unmanipulated control days, broods received an average of 13.4 prey items/h from male carers (7.0 SD, range = 5.6–33.7). Following the removal of 1–3 such carers, broods received 19% less food/h for each carer removed, on average (regression estimate [± SE] = −5.3±1.6; 43% variance explained; permutation test *P* < 0.001; Figure 1a). As a consequence, when carers were removed, broods received an average of 8.8 prey items/h from male carers (5.0 SD, range = 3.9–19.3), 34% less than on control days.

Groups used in the brood size manipulations provisioned an average of 12.7±6.8 (SD) items/h on control days (range = 5.2–27.5), a comparable rate to those observed on control days in the carer removal experiment (*T*_31_ = −0.29, *P* = 0.78). Carers were highly responsive to the addition of 1–3 nestlings, with broods receiving prey 18% more frequently with every nestling added, on average (regression estimate [± SE] = 2.6±1.1; 21% variance explained; permutation test *P* < 0.001; Figure 1b). As a consequence, on experimental days, broods received an average of 16.9 prey items/h from male carers (±9.8 SD, range = 5.4–39.9), a 33% increase from control days. Surprisingly, the magnitude of this effect led each nestling to receive an extra 0.7 prey items/h on average following brood size enhancement than on control days (±0.7 SD, range = −0.4 to +2.2).

To elucidate whether territory quality contributed significantly to the apparently causal effects of carer numbers on brood provisioning rates, we investigated the relative impacts of control versus experimental numbers of male carers on per capita rates of nestling food acquisition on manipulation days. The rate at which each nestling received food was determined by the number of male carers remaining (*F*_1,13_ = 5.61, *P* = 0.035, estimate [± SE] = 0.56±0.24) and not the control number (*F*_1,13_ = 1.84, *P* = 0.20, estimate [± SE] = −0.28±0.21). These results suggest that any effect of territory quality on the number of male carers does not confound estimates of carer contributions in this system, corroborating a causal positive relationship between male carer number and rates of offspring food acquisition.

### Comparing the experimental approaches

**Figure 2:**
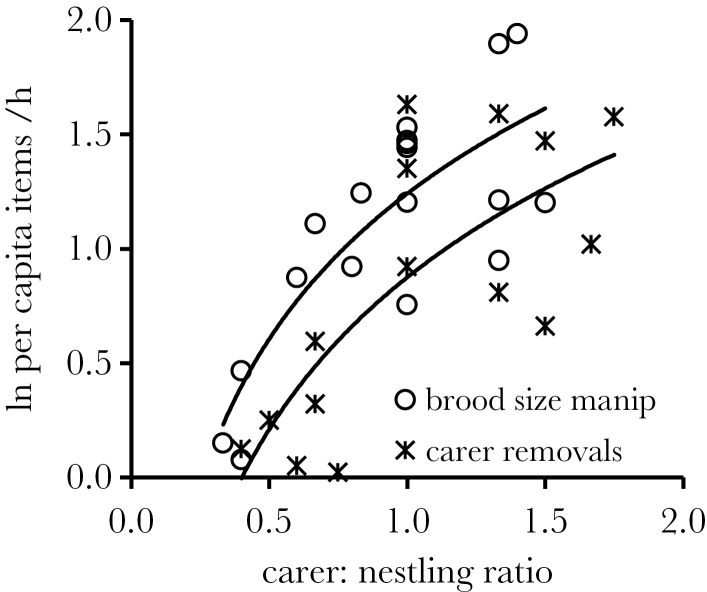
Comparing the outcome of the 2 approaches. For a given carer:nestling ratio, per capita nestling provisioning rates were higher following brood enhancement (upper line) than carer removal (lower line). Figure shows ln-transformed raw data and best-fit logarithmic functions.

Despite equalizing carer:nestling ratios in the 2 experiments, equivalent relationships between carer:nestling ratios and per capita nestling provisioning rates were not observed. Although the slope gradients of these relationships were equivalent across the 2 experiments (interaction term: GLM *F*_1,26_ = 0.01, *P* = 0.95), the elevation was significantly different (treatment effect: *F*_1,18_ = 11.37, *P* = 0.003; Figure 2). Specifically, after controlling for significant positive effects of carer:nestling ratios (*F*_1,30_ = 44.77, *P* < 0.001, estimate [±SE] = 0.93±0.14), individual nestlings received prey items 47% more frequently on experimental days of the brood size manipulations compared with those of male carer removals. Put another way, each nestling received prey significantly more frequently with a carer:nestling ratio of 1:1 that was generated by enlarging brood size (e.g., from 3 to 5 in a group of 5) than was generated by removing carers (e.g., from 5 to 3 in a brood of 3).

### Explaining the differences

**Figure 3:**
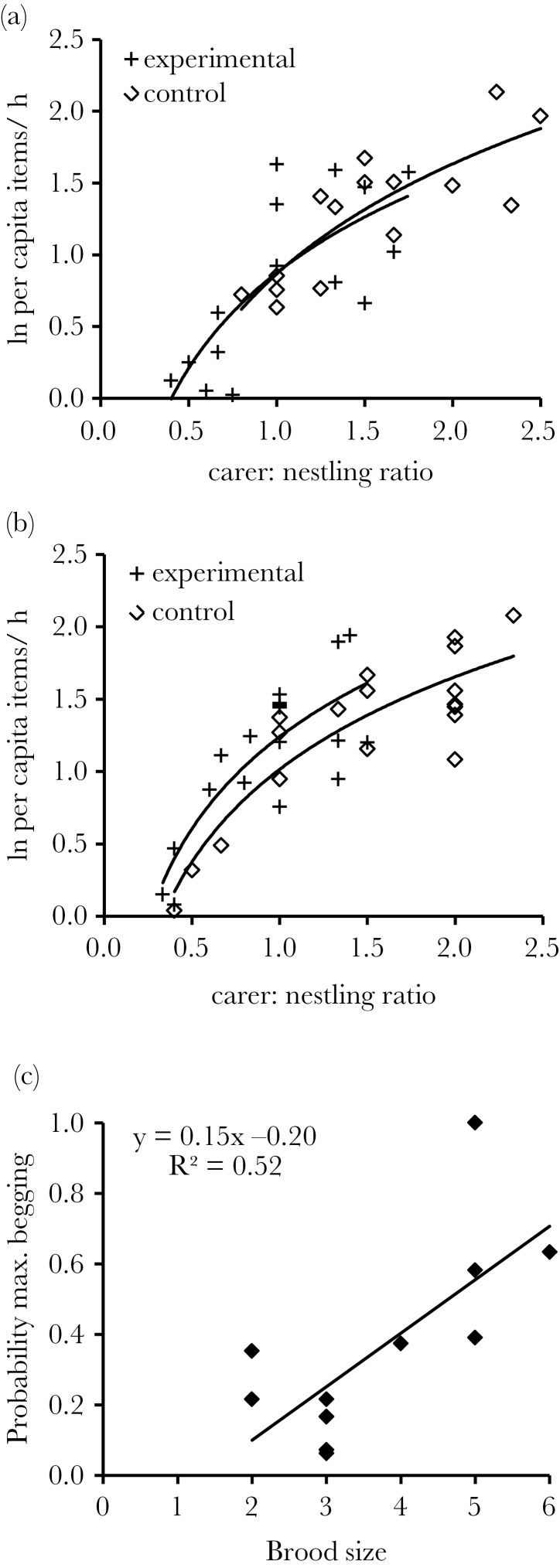
Explaining the differences. (a) Carer removal: Carer:nestling ratios had substantial, although diminishing, positive effects on per capita nestling provisioning rates (ln-transformed), but the functions of the relationship were similar on control days (solid line) versus experimental days (dashed line) (control days: *y* = 1.10ln[*x*] + 0.87, *R* ^2^ = 0.69; experimental days: *y* = 0.96ln[*x*] + 0.87, *R* ^2^ = 0.56). (b) Brood enhancement: Carer:nestling ratios again had substantial, although diminishing, positive effects on (ln-transformed) per capita nestling provisioning rates. However, in this case, the intercepts of slope functions significantly differed, with nestlings receiving more food for a given carer:nestling ratio on experimental days (dashed line) compared with control days (solid line) (control days: *y* = 0.92ln[*x*] + 1.01, *R* ^2^ = 0.78; experimental days: *y* = 0.92ln[*x*] + 1.24, *R* ^2^ = 0.66). (c) The probability of experiencing maximally begging broods increased as a function of its size, after controlling for rates and timing of nestling food acquisition. This is supportive of scramble competition and suggests that carers will more commonly encounter maximal begging nestlings following brood size enhancement. Figures (a) and (b) show raw data and best-fit logarithmic functions, where (c) shows raw means for each brood and best-fit linear function.

The difference in provisioning rates arising from the 2 experiments could derive from disruptive effects of carer removals. If carer removals were disruptive we would predict that 1) after controlling for variation in carer:nestling ratios, per capita nestling provisioning rates would be significantly reduced on removal days compared with control days and 2) the relationship between carer:nestling ratios and per capita nestling provisioning rates would be significantly flatter on manipulation days than control days. Neither was true (Figure 3a). First, after controlling for substantial positive, although diminishing, effects of increasing carer:nestling ratios on per capita nestling provisioning rates (log function: REML *F*_1,19_ = 31.60, *P* < 0.001, estimate [± SE] = 1.04±0.18), there was no evidence of an additional treatment effect (*F*_1,26_ = 0.01, *P* = 0.92). Second, the interaction between treatment and carer:nestling ratio on per capita nestling provisioning rates was nonsignificant (*F*_1,15_ = 0.48, *P* = 0.50), indicating that the relationships between carer:nestling ratio and per capita nestling provisioning rates are equivalent on control and removal days. These findings suggest that carer removals do not confound carer effects in this system and so are unlikely to explain differences in the results obtained using the 2 experimental techniques.

An alternative possibility is that differences are generated from the brood size manipulation experiment. In support, significant differences were found between per capita nestling provisioning rates on control versus experimental days in the brood size manipulation. As expected, carer:nestling ratios had a significant effect on per capita nestling provisioning rates (REML *F*_1,28_ = 53.46, *P* < 0.001, estimate [± SE] = 0.85±0.11), and the interaction between treatment and carer:nestling ratios was nonsignificant (*F*_1,16_ = 0.02, *P* = 0.90). However, in contrast to the carer removal experiment, treatment remained a significant predictor of nestling provisioning rates after controlling for carer:nestlings ratios, with each nestling being provisioned 21% more frequently by male carers on experimental days than expected given their carer:offspring ratio (*F*_1,33_ = 9.34, *P* = 0.004, estimate [± SE] = 0.19±0.06; Figure 3b).

A likely mechanism for the relative increases in provisioning rates on experimental days of the brood size manipulation is scramble competition (Royle et al. 2002). During provisioning, carers encountered maximally begging nestlings on 30% of visits. The probability that maximally begging nestlings would be encountered decreased with increasing per capita nestling food delivery rates during the previous hour ($\chi_{1}^{2}$ = 12.13, *P* < 0.001, estimate [±SE] = −0.15±0.042) and time since last feed (linear effect: $\chi_{1}^{2}$ = 18.11, *P* < 0.001, estimate [±SE] = 0.051±0.011; quadratic effect: $\chi_{1}^{2}$ = 9.00, *P* < 0.001, estimate [±SE] = −0.00021±0.000070). These results verify that short-term changes in hunger (even within an hour) are sufficient to generate increased begging. After controlling for these hunger-effects, we found additional significant effects of brood size (GLMM: $\chi_{1}^{2}$ = 9.90, *P* < 0.001, estimate (±SE) = 0.63±0.10, Figure 3c), but not carer numbers ($\chi_{1}^{2}$ = 0.23, *P* = 0.63, estimate [±SE] = 0.06±0.12). These results support the hypothesis that begging intensity is influenced by competition among nest mates (Royle et al. 2002) and might help to explain why enlarged broods receive food faster than expected after controlling for their change in carer:offspring ratios.

## DISCUSSION

Our key aim was to test the effects of additional carers on a salient component of success in chestnut-crowned babblers. Provisioning rate is a key component of success in chestnut-crowned babblers: The rate of food acquisition is the primary determinant of prey biomass received by offspring (Browning, Young, et al. 2012) and nestling starvation is the primary determinant of nestling fledging failure (Russell et al. 2010). Using 2 independent experimental methods, we provide strong supporting evidence that having additional carers has a direct positive effect on offspring provisioning rates in a cooperative breeder. More specifically, the removal of carers caused reductions in rates of nestling food acquisition that could not easily be explained by territory quality or any potential disruptive effects of the experimental manipulation (e.g., to group dynamics or foraging efficiency), while the addition of nestlings indicated that carers are responsive to changes in brood demand. Surprisingly, however, the 2 techniques did not generate quantitatively equivalent results: Each nestling was provisioned more often following brood size enhancement than following male carer removal, despite equal carer:nestling ratios in each. Finally, nestling begging intensity appears to be influenced by both hunger and brood size, suggesting that scramble competition might mediate the greater provisioning rates following brood enhancement.

Arguably the most significant problem with demonstrating and quantifying the causal effects of additional carers on parameters of success in cooperative breeders is variation in territory quality. This is because high-quality habitats can independently have both high breeding success and many carers (Dickinson and Hatchwell 2004). Manipulations of brood size (Clutton-Brock et al. 2001; Russell et al. 2008; Koenig and Walters 2012) (or begging intensity; Wright 1998; MacGregor and Cockburn 2002; McDonald et al. 2009) confirm that carers are responsive to brood demand and, by extension, contribute to the growth and survival of offspring, but they cannot obviously quantify the confounding influence of territory quality. By contrast, carer removal experiments can be more illuminating in this regard because the confounding magnitude of territory quality versus the true magnitude of the carer effect will be borne in the effect sizes of the control versus manipulated numbers of carers on the response term. To illustrate, at the extremes, a sole effect of control carer number on the brood provisioning rates during experimental days would be supportive of a confounding effect of territory quality, whereas a sole effect of experimental carer numbers would be indicative of a causal carer effect. We found support for the latter. This makes sense in chestnut-crowned babblers due to their plural breeding system (Portelli et al. 2009) and weak territoriality (Sorato et al. 2015), which permit variably sized units to share the same habitat.

Despite the illustrated benefits, removing carers from groups might induce spurious changes to provisioning if, following removal, remaining carers have reduced foraging efficiency or partake in renewed disputes of dominance (Cockburn 1998). We suggest that such an effect would flatten the relationship between carer:offspring ratio on experimental relative to control days. We found no evidence of this. In addition, we found no compelling evidence to suggest that reduced provisioning following carer removal arose as a consequence of other potential confounds (e.g., less demand from nestlings). We found here that nestling begging intensity is sensitive to per capita nestling provisioning rates over the previous hour as well as time since last feed. This sensitivity, combined with the fact that broods were subject to fewer carers for a day before observation, makes it highly unlikely that begging intensity did not rise following carer removal. Furthermore, it is unlikely that remaining carers compensated by delivering larger prey items, as there is no evidence for a relationship between carer number and the size of the single prey item delivered (Browning, Young, et al. 2012). Finally, we do not expect the breeding female would compensate for the provisioning care lost by removed individuals as she responds insufficiently to variation in helper number (Browning, Young, et al. 2012): The breeding female increases her provisioning rate only by circa 0.3 feeds/h for every incremental reduction in carer number, whereas following carer removals, broods generally received 2–4 fewer feeds/h/male carer lost. Thus, we found no obvious evidence to suggest that removing carers introduces significant confounds to the aims addressed.

Although we suggest that manipulating carer number provides significant insight into causality and magnitude of the effect carers have on offspring, the results of the brood size manipulation were also illuminating. Most notably, nestlings received prey at a significantly elevated rate following brood enlargement relative to their carer:nestling ratio. One possibility is that these differences are artificial and do not translate into differences in biomass delivered (i.e., if babblers delivered more, smaller prey items to larger broods). However, this is unlikely as provisioning rate and prey size are not related (Browning, Young, et al. 2012). Because nestlings should be equally hungry under the same carer:nestling ratio, whether derived from carer or brood size manipulations, an alternative hypothesis for the relatively greater provisioning rates observed following brood enlargement is scramble competition (Royle et al. 2002). In support, we found begging intensity to be strongly influenced by brood size, even after controlling for obvious hunger correlates (i.e., time since last feed and per capita nestling provisioning rate during the previous hour). Although demonstrating scramble competition definitively is challenging, it is expected in species such as babblers in which nestling starvation is high and prey is delivered singly (Royle et al. 2002). Either way, our results suggest that brood size manipulations might introduce hitherto unconsidered confounds when used to quantify the magnitude of carer influences, particularly when scramble competition introduces exaggerated responses.

Despite the support for scramble competition here, it is difficult to envisage it accounting functionally for the difference in results arising from the 2 experiments. Assuming both experiments generated increased begging, which seems inevitable given that both should generate hungrier nestlings and begging intensity is highly sensitive to short-term variation in prey delivery, why should carers only respond to increased begging when it reflects both increased hunger and competition? Unless responses are only generated once a threshold level of begging intensity is surpassed, and to exceed such thresholds requires combined pressures, another explanation is required to answer this question more fully. Theoretically, carers should not respond to increased brood demand when the costs of doing so accelerate (Johnstone 2011); that carers do not obviously respond to the removal of co-carers is supportive. And yet, carers do respond to increased brood size. One explanation for this apparent paradox is that carers may respond positively to brood enhancement experiments because larger broods offer greater fitness returns per unit investment (i.e., the accelerating costs are offset by higher benefits; McAuliffe et al. 2015). This hypothesis suggests that carers respond more to variation in brood size because of changes to available fitness rather than changes to brood demand per se.

More generally, quantifying the effects of carer numbers on parameters of success lends itself to better understanding the evolution of cooperative breeding systems. Despite the power of manipulative experiments in elucidating such effects, few studies have adopted such an approach, partially because confusion surrounds the most appropriate means of doing so. Here, we argue both group size and brood size manipulations are informative, if not equivalent. That being said, when determining which method is most appropriate, a few considerations need to be made. First, the carer removal approach is the only one with the potential to reveal the precise function of any relationship between carer number and response parameters. Second, despite other potential confounds (e.g., Russell et al. 2007; Cockburn et al. 2008), the most significant problem in quantifying the effects carers have on offspring is likely to stem from the autocorrelation between territory quality and carer number (Brown 1987; Dickinson and Hatchwell 2004). As a result, an obvious way of controlling for this is to manipulate carer number experimentally and then fit both the premanipulated and postmanipulated number of carers in the analysis. Third, that carer removals have the potential to introduce further confounds (Cockburn 1998) are as yet unsubstantiated, and we found no evidence that this was the case here (see also Liebl et al. 2016). On the contrary, we found evidence to suggest that brood size manipulations might introduce confounds by influencing the degree of scramble competition among nest mates. As a result, we suggest that brood size manipulations, and presumably by extension manipulation of begging intensity, are better suited to testing carer responses to offspring demand and understanding carer provisioning rules.

In conclusion, consistent with a meta-analysis in biparental care systems (Harrison et al. 2009), different experimental approaches meant to test for an effect of carers in cooperative breeders generate quantitatively contrasting results. In this study, carers responded more to manipulated brood sizes than would be expected given the carer:nestling ratios. Although this difference appears to be mediated in part by scramble competition, we predict that the ultimate reason is due to increasing potential fitness benefits, but this requires further study. Although not equivalent, manipulations of carer:offspring ratios through carer removal and brood size changes represent complimentary approaches with the potential of yielding greater insights into (allo-)parental care strategies. For example, we showed that the effects of additional carers are additive and uninfluenced by territory quality using carer removals, whereas brood size manipulation complimented these findings by confirming that carers were responsive to changes in brood size. Therefore, despite illuminating mechanistic differences, both methods indicated a causal impact of increasing carer numbers relative to offspring number on offspring food acquisition rates in this species, confirming that additional carers are adaptive in chestnut-crowned babblers.

## FUNDING

The project was funded by grants to A.F.R. from the Australian Research Council Discovery Grant (A.F.R.: DP0774080 and DP1094295) and Natural Environment Research Council (A.F.R.: NE/K005766/1).
